# Supplementary material for: Lipidomic QTL in Diversity Outbred mice identifies a novel function for α/β hydrolase domain 2 (Abhd2) as an enzyme that metabolizes phosphatidylcholine and cardiolipin
Source: bioRxiv. 2023 Mar 24:2023.03.23.533902. Preprint. [Version 1] doi: 10.1101/2023.03.23.533902 (PMC10055419; doi:10.1101/2023.03.23.533902)
Supplement: Supplement 1 [file NIHPP2023.03.23.533902v1-supplement-1.pdf]

bioRxiv preprint doi: https://doi.org/10.1101/2023.03.23.533902; this version posted March 24, 2023. The copyright holder for this preprint (which was not certified by peer review) is the author/funder, who has granted bioRxiv a license to display the preprint in perpetuity. It is made available under aCC-BY 4.0 International license.

Supporting Information – Supplemental Figure Captions

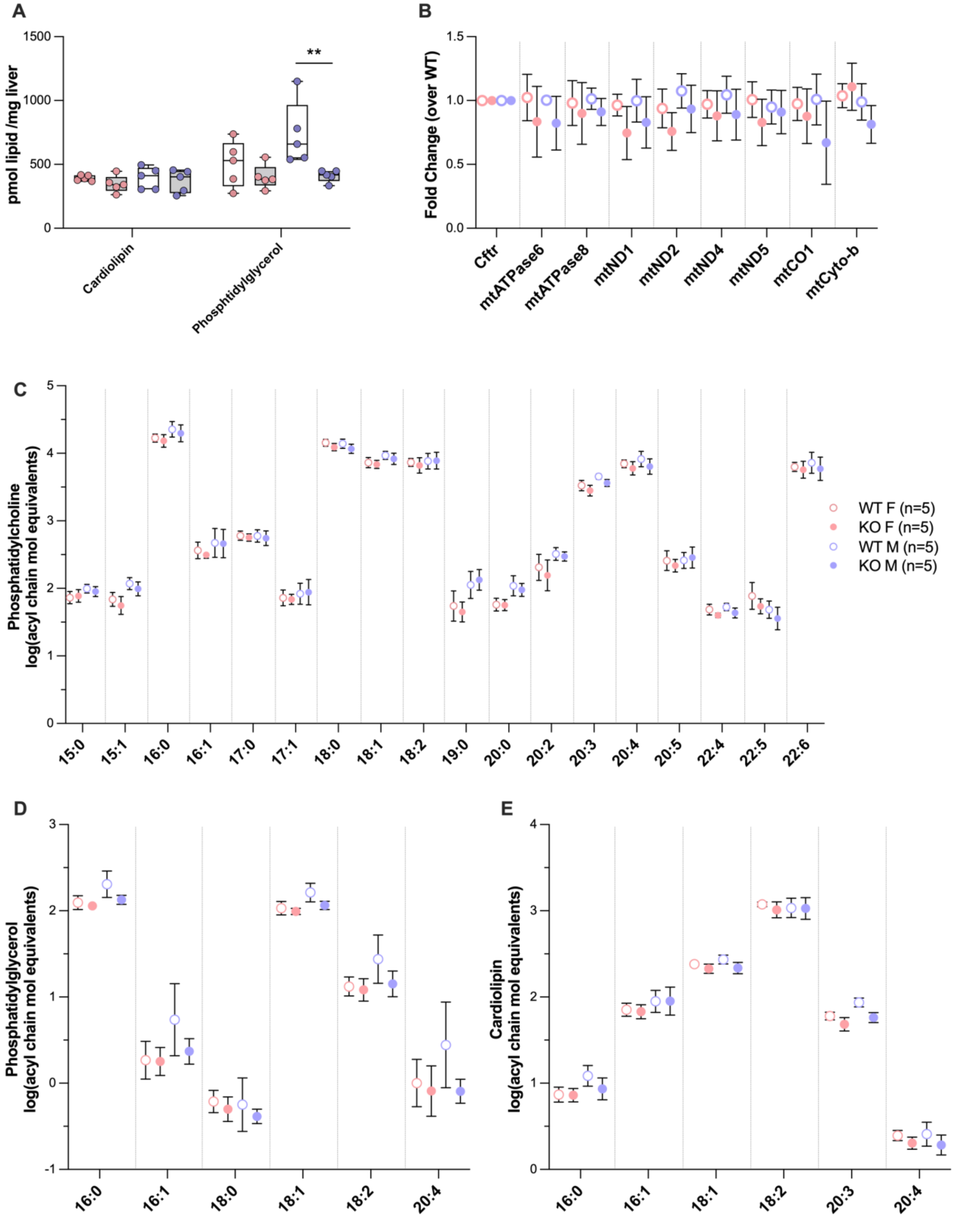

Figure S1. ***Abhd2*** **deletion decreased hepatic phosphatidylglycerol concentrations but did not alter mitochondrial gene expression or mitochondrial lipid acyl chain compositions**

bioRxiv preprint doi: https://doi.org/10.1101/2023.03.23.533902; this version posted March 24, 2023. The copyright holder for this preprint (which was not certified by peer review) is the author/funder, who has granted bioRxiv a license to display the preprint in perpetuity. It is made available under aCC-BY 4.0 International license.

(**A**) Despite significant reductions in several cardiolipin species in male mice, total hepatic cardiolipin levels in male and female mice did not differ by genotype. However, total phosphatidylglycerol concentrations were decreased Abhd2^{KO} mice compared to WT males ($p<0.01$). (**B**) Mitochondrial gene expression, measured as a proxy for mitochondrial number, was not different by sex or genotype. Neither genotype nor sex affected fatty acyl composition of PC, PG or CL in the livers of HF/HS-fed mice. (**C**) The hepatic phosphatidylcholine landscape was diverse and were primarily comprised of acyl chains of C16 or C18 in length and were saturated or monounsaturated. (**D**) Phosphatidylglycerols were equally represented by fatty acyl lengths of C16 and C18 and contained 0 or 1 double bond. (**E**) Cardiolipins were highly represented by linoleate, with C18 being 95% of acyl lengths and 98% of CLs containing 1 or more double bonds.

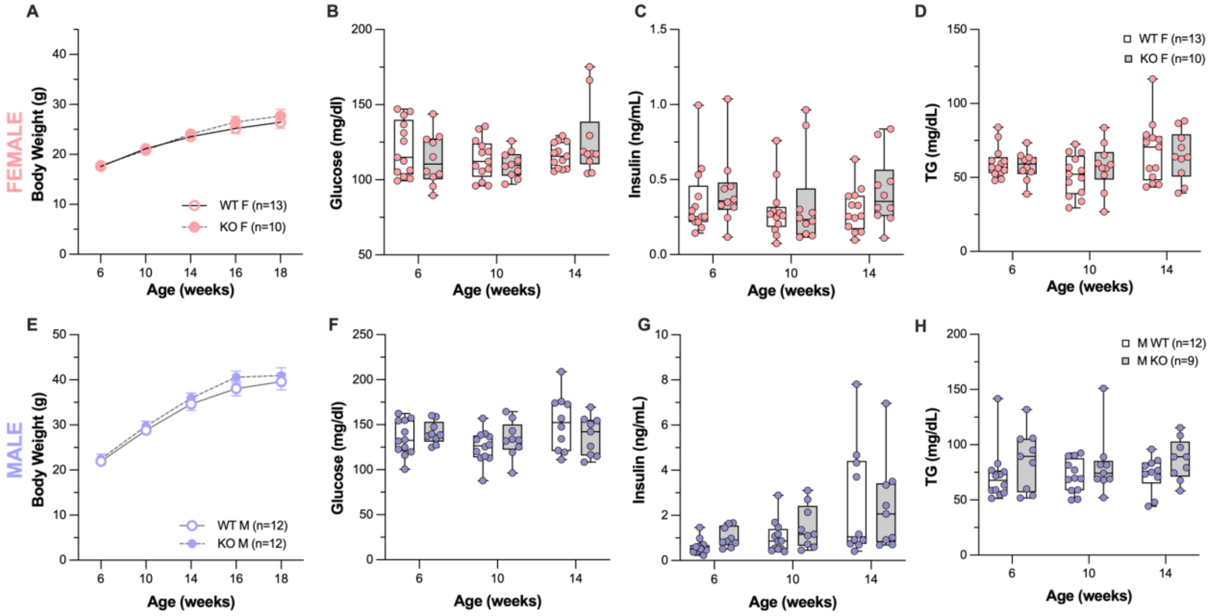

Figure S2. Whole-body deletion of *Abhd2* did not alter growth rates nor fasting blood profiles in C57Bl6/J mice.
Abhd2^{KO} female (**A**) and male (**E**) mice showed similar growth curves to WT mice. Fasting glucose (**B, F**), insulin (**C, G**), and triglycerides (**D, H**) did not differ by genotype.

bioRxiv preprint doi: https://doi.org/10.1101/2023.03.23.533902; this version posted March 24, 2023. The copyright holder for this preprint (which was not certified by peer review) is the author/funder, who has granted bioRxiv a license to display the preprint in perpetuity. It is made available under aCC-BY 4.0 International license.

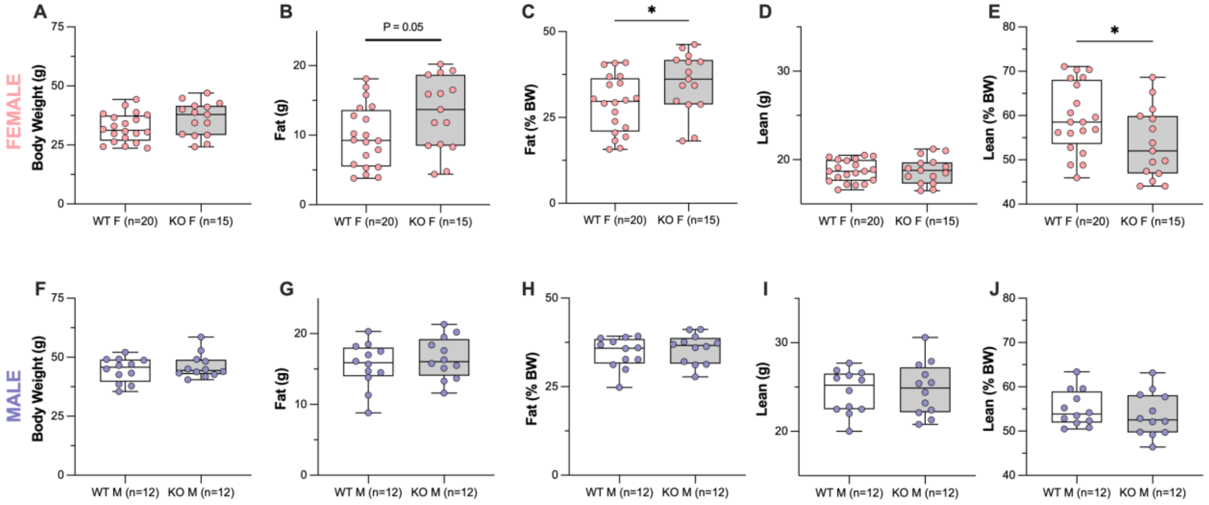

Figure S3. Loss of *Abhd2* altered body compositions of female mice by increasing fat mass as measured by DEXA.

Body compositions of mice were measured at ~24 weeks of age by DEXA. (**A**) Body mass of female mice were not significantly different. Fat mass, both as total weight (**B**) and %body weight (**C**) increased in Abhd2^{KO} female mice. Lean mass weight (**D**) did not change with genotype in females, but lean mass as %body weight (**E**) was reduced in Abhd2^{KO} female mice. Male mice were not different in total body weight, fat, nor lean mass (**F-J**). *p<0.05

bioRxiv preprint doi: https://doi.org/10.1101/2023.03.23.533902; this version posted March 24, 2023. The copyright holder for this preprint (which was not certified by peer review) is the author/funder, who has granted bioRxiv a license to display the preprint in perpetuity. It is made available under aCC-BY 4.0 International license.

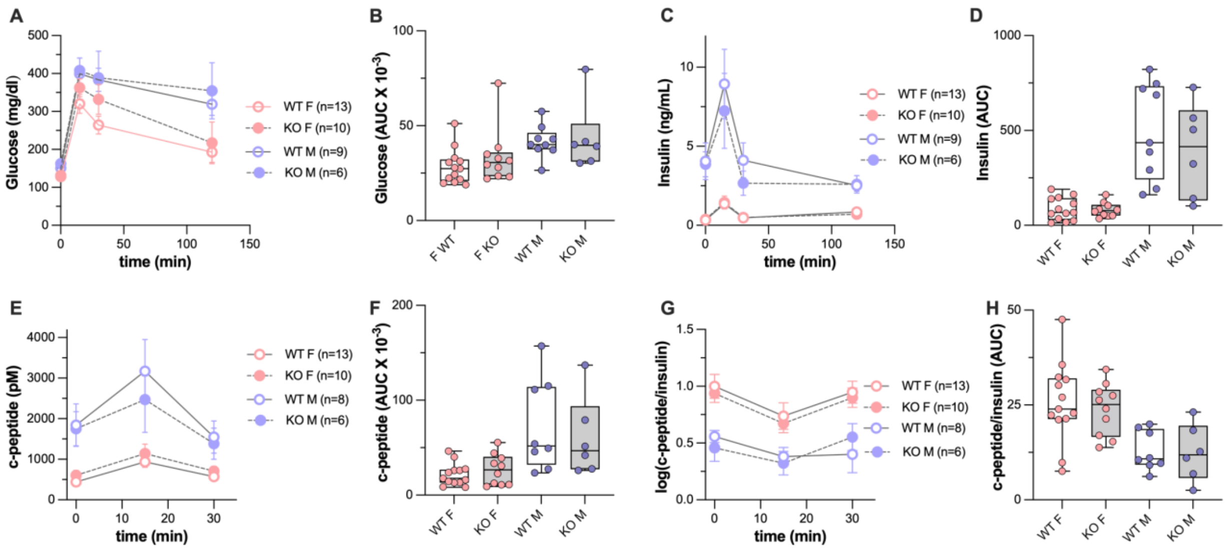

Figure S4. Assessment of insulin action by oral glucose tolerance test (oGTT) elicited similar responses between genotypes of the same sex

(**A**) Female Abhd2^{KO} mice showed a trend for increased plasma glucose at 15 and 30-minute timepoints during the oGTT. Male Abhd2^{KO} mice were not different. (**B**) Area under the curve (AUC) for plasma glucose during the oGTT did not differ by genotype. (**C**) Plasma insulin response to glucose stimulation were the same for genotypes of each sex, with all mice returning to baseline within two hours of receiving the glucose bolus. (**D**) Insulin curve AUCs were not different. (**E**) C-peptide, a marker of insulin secretion, was the same for genotypes of each sex during the oGTT, with no difference in AUC (**F**). (**G**) The C-peptide/insulin ratio, used as a surrogate for insulin clearance, were not different at 0, 15, and 30 minutes. (**H**) AUCs for C-peptide/insulin ratio were similar between genotypes of the same sex.

bioRxiv preprint doi: https://doi.org/10.1101/2023.03.23.533902; this version posted March 24, 2023. The copyright holder for this preprint (which was not certified by peer review) is the author/funder, who has granted bioRxiv a license to display the preprint in perpetuity. It is made available under aCC-BY 4.0 International license.

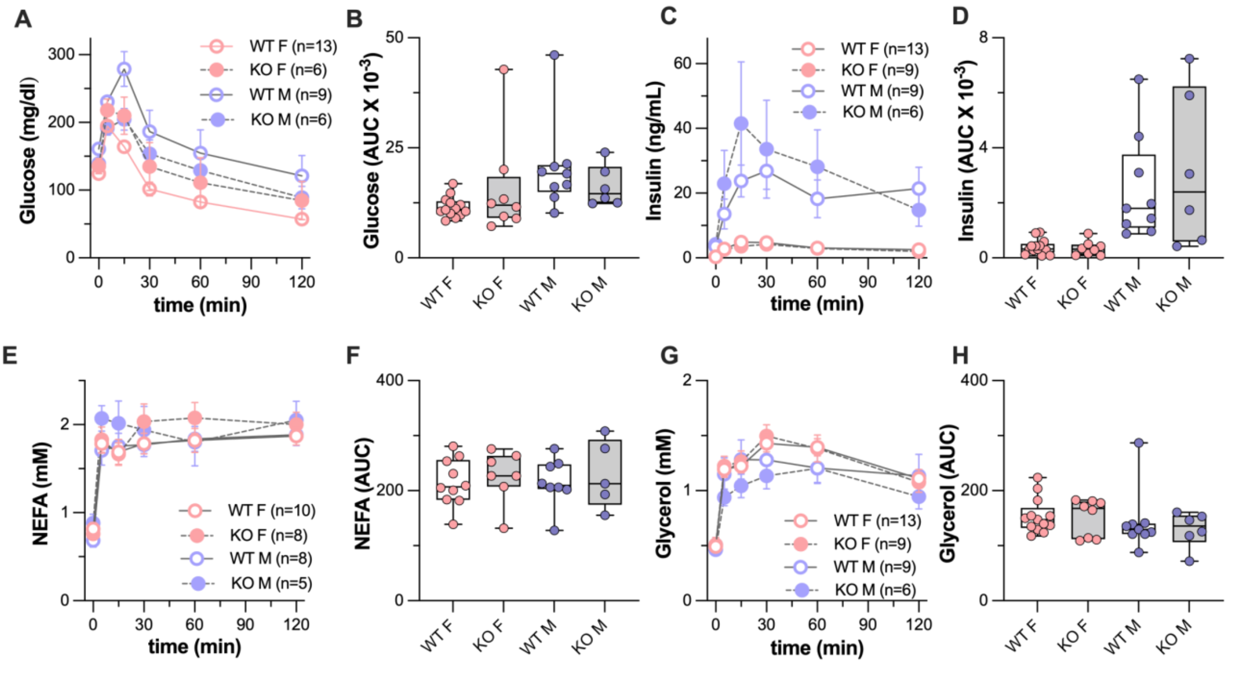

Figure S5. β3-adregeneric receptor agonist stimulation failed to produce a physiologic response in *Abhd2* KO mice

Plasma glucose concentrations at various time points (**A**) and total AUC for glucose (**B**) during β3-adregeneric receptor agonist stimulation was not different in male or female Abhd2^{KO} mice. Plasma insulin concentrations (**C**) and total AUC for insulin (**D**) during the B3TT were the same for genotypes of each sex. Non-esterified fatty acid (NEFA) concentration (**E**) and total AUC for NEFA (**F**), and glycerol concentration (**G**), and AUC for glycerol (**H**) during the β3TT did not different for Abhd2^{KO} female or male mice.

bioRxiv preprint doi: https://doi.org/10.1101/2023.03.23.533902; this version posted March 24, 2023. The copyright holder for this preprint (which was not certified by peer review) is the author/funder, who has granted bioRxiv a license to display the preprint in perpetuity. It is made available under aCC-BY 4.0 International license.

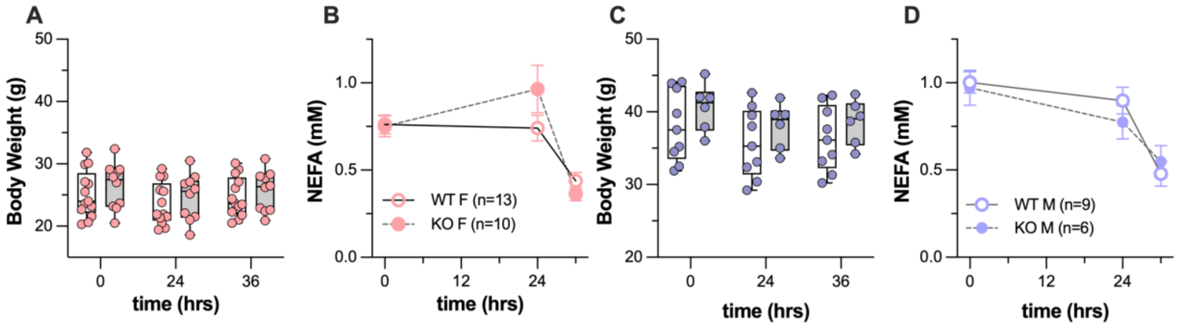

Figure S6. Loss of *Abhd2* does not alter the physiological response to prolonged fasting or refeeding.

Following a 24-hr fast, female mice averaged a 1.7 ± 0.9 gm weight loss and an average 1.1 ± 0.1 gm weight gain following the 6-hour refeed period and were not different for Abhd2^{KO} versus WT mice (**A**). Plasma NEFAs, measured before and after prolonged fast, were similar between genotypes (**B**). Male mice lost 2.5 ± 0.2 gm with prolonged fasting and regained 0.6 ± 0.1 gm following refeeding, and were not different between genotypes (**C**). Plasma NEFAs of male mice during the fast/refeed protocol did not differ by genotype (**D**).

bioRxiv preprint doi: https://doi.org/10.1101/2023.03.23.533902; this version posted March 24, 2023. The copyright holder for this preprint (which was not certified by peer review) is the author/funder, who has granted bioRxiv a license to display the preprint in perpetuity. It is made available under aCC-BY 4.0 International license.

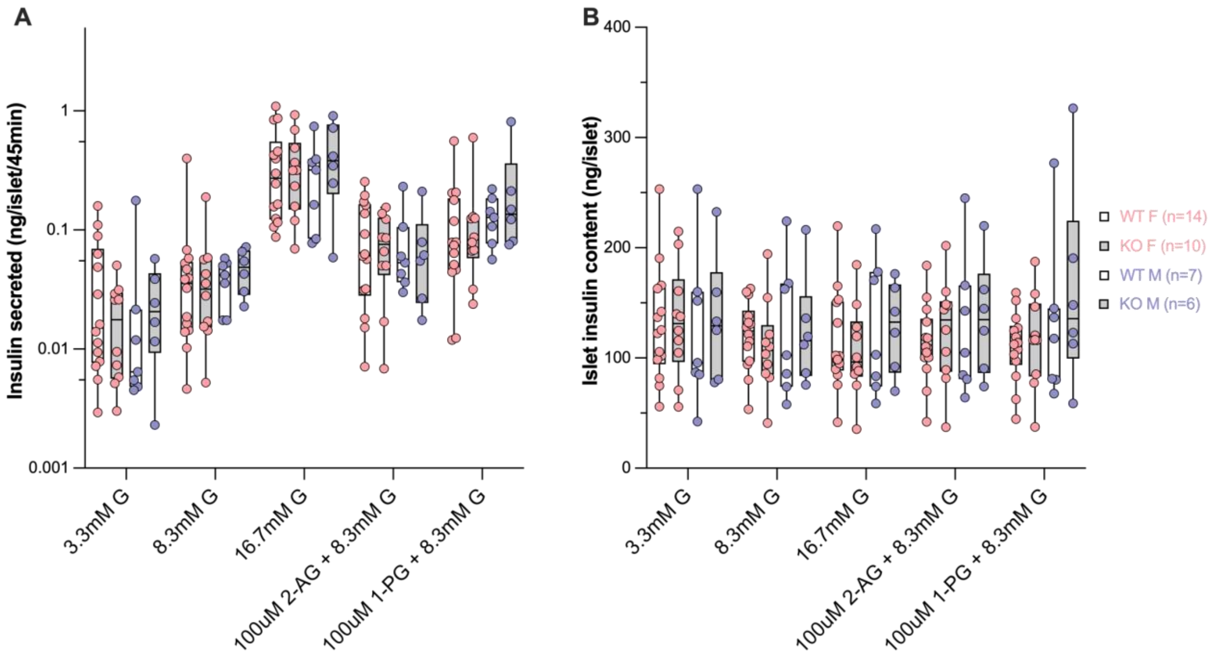

Figure S7. Loss of *Abhd2* did not alter insulin secretion in response to glucose or monoacylglycerol

Insulin secretion in response to varying glucose concentration, or two different monoacyl-glycerols (2-AG or 1-PG) (**A**) or total islet insulin content (**B**) remained unchanged in cultured islets from female and male Abhd2^{KO} versus WT mice.

bioRxiv preprint doi: https://doi.org/10.1101/2023.03.23.533902; this version posted March 24, 2023. The copyright holder for this preprint (which was not certified by peer review) is the author/funder, who has granted bioRxiv a license to display the preprint in perpetuity. It is made available under aCC-BY 4.0 International license.

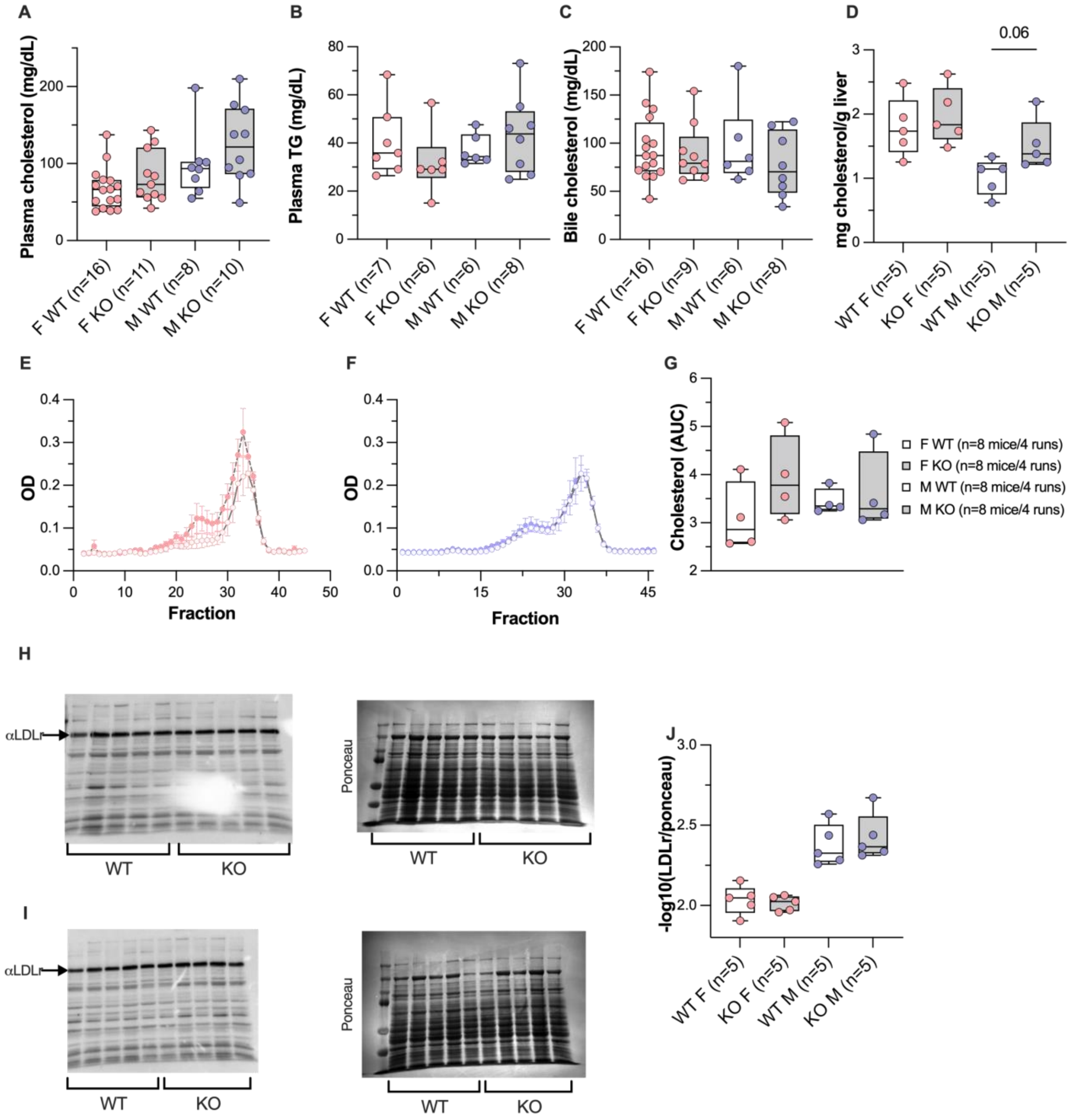

Figure S8. Loss of Abhd2 exerts a subtle influence on whole-body cholesterol metabolism.

Total plasma cholesterol (**A**) and triglycerides (**B**), biliary cholesterol (**C**), and hepatic cholesterol (**D**) in female and male Abhd2^{KO} versus WT mice. Male Abhd2^{KO} mice showed a small increase in hepatic cholesterol (p = 0.06). Plasma cholesterol lipoproteins were separated by FPLC and assayed for cholesterol in female (**E**) and male (**F**) mice. Total

bioRxiv preprint doi: https://doi.org/10.1101/2023.03.23.533902; this version posted March 24, 2023. The copyright holder for this preprint (which was not certified by peer review) is the author/funder, who has granted bioRxiv a license to display the preprint in perpetuity. It is made available under aCC-BY 4.0 International license.

AUC for cholesterol in all FPLC fractions (**G**). Liver from female (**H**) and male (**I**) mice were analyzed for LDL-receptor (LDLR) protein content by immunoblot. (**J**) Quantitation of LDLR protein abundance was not different between genotypes of the same sex.
